# Immunoregulation by *Taenia crassiceps* and Its Antigens

**DOI:** 10.1155/2013/498583

**Published:** 2012-12-27

**Authors:** Alberto N. Peón, Arlett Espinoza-Jiménez, Luis I. Terrazas

**Affiliations:** Unidad de Biomedicina, Facultad de Estudios Superiores Iztacala, Universidad Nacional Autónoma de México, Avenida De los Barrios 1, Los Reyes Iztacala, 54090 Tlalnepantla, MEX, Mexico

## Abstract

*Taenia crassiceps* is a cestode parasite of rodents (in its larval stage) and canids (in its adult stage) that can also parasitize immunocompromised humans. We have studied the immune response elicited by this helminth and its antigens in mice and human cells, and have discovered that they have a strong capacity to induce chronic Th2-type responses that are primarily characterized by high levels of Th2 cytokines, low proliferative responses in lymphocytes, an immature and LPS-tolerogenic profile in dendritic cells, the recruitment of myeloid-derived suppressor cells and, specially, alternatively activated macrophages. We also have utilized the immunoregulatory capabilities of this helminth to successfully modulate autoimmune responses and the outcome of other infectious diseases. In the present paper, we review the work of others and ourselves with regard to the immune response induced by *T. crassiceps* and its antigens, and we compare the advances in our understanding of this parasitic infection model with the knowledge that has been obtained from other selected models.

## 1. Introduction

Helminth parasites have developed complex and versatile mechanisms to evade the immune responses of their hosts, utilizing immunoregulatory strategies to avoid immune effector mechanisms. In general, these processes are necessary for the parasites to complete their long life cycles [1] and/or to favor host survival [2]. Despite their great evolutionary divergence and variety of stages, life cycles, and pathogenic and invasive mechanisms, helminths have developed similar strategies and induce strikingly similar immune responses, which have been called “stereotypical Th2-type immune responses.” However, there are differences in the immune responses evoked by distinct helminths, mainly with regard to leukocyte involvement and the roles of these cells [3]. 

The stereotypical Th2 response induced by helminth parasites is characterized by the secretion of high levels of anti-inflammatory cytokines such as interleukin-6 (IL-6), IL-9, IL-10, IL-25, IL-33, and transforming growth factor-*β* (TGF-*β*), but the main cytokines are IL-4 and IL-13 [4]. As a consequence and/or origin of this cytokine secretion, there are alterations in leukocyte recruitment and activation, such as high levels of CD4+ T lymphocytes differentiated into Th2 and T regulatory (Treg) subsets, the recruitment and activation of immunoglobulin G1 (IgG1)- and IgE-producing B cells, eosinophilia, basophilia, and mastocytocis [4, 5]. Interestingly, an immature dendritic cell (iDC) phenotype with a Th2-driving ability and huge populations of alternatively activated macrophages (AAMs) with the ability to suppress lymphocyte proliferation can also be found within this response [3, 5, 6]. Furthermore, another characteristic of Th2 responses is the suppression of the immune response to bystander antigens, which may compromise the effectiveness of vaccination [7] and alter the immune response to several other antigens, even autoantigens. 

It is commonly accepted that most of these changes in leukocyte phenotype and activation, as well as in the induction of the inflammatory milieu, are dependent upon the ability of the parasite to excrete/secrete antigens with immunoregulatory properties [8–12]. Many research teams [13–15], including ours [16, 17], have used these Th2 responses elicited by helminths and their antigens to control autoimmune disease development as well as to alter the outcome of other infectious diseases [18].

## 2. The Immune Response to Experimental *T. crassiceps *Infection: Th1/Th2 Balance and Susceptibility

*T. crassiceps* is a helminth parasite (class Cestoda) that can be found in its adult form within the small intestine of canids, whereas the main larval stage (metacestode) can be found in the muscles, peritoneal, and pleural cavity of rodents. *T. crassiceps* metacestodes can also parasitize immunocompromised human patients with cancer [19], human immunodeficiency virus and hepatitis C virus [20]. In addition, this parasite can infect perfectly healthy patients, although only one case has been reported [21]. An interesting feature of *T. crassiceps* is its ability, or evolutive advantage, to reproduce asexually through budding at the larval stage. This characteristic permits the larval stage to maintain and colonize its hosts for long periods of time; thus, after the intraperitoneal inoculation of a few parasites (10 to 20 metacestodes), hosts can harbor hundreds of parasites 6–8 weeks later. This feature has been useful for maintaining the parasite at the larval stage in the laboratory via passage from mouse to mouse through intraperitoneal injections, and these animals are also important sources of antigens that have been utilized for immunodiagnostic tests for cysticercosis [22]. Additionally, the fact that the larval stage of the parasite is innocuous for humans is important; although its macroscopic size facilitates the accumulation of an acute parasite burden, the parasite does not kill the host and is able to cause chronic infections with a minimum amount of damage in mice. Furthermore, the results are very reproducible. All of these features confer many advantages on this model for laboratory work and even for the development of vaccine strategies [23]. 

**Figure 1:**
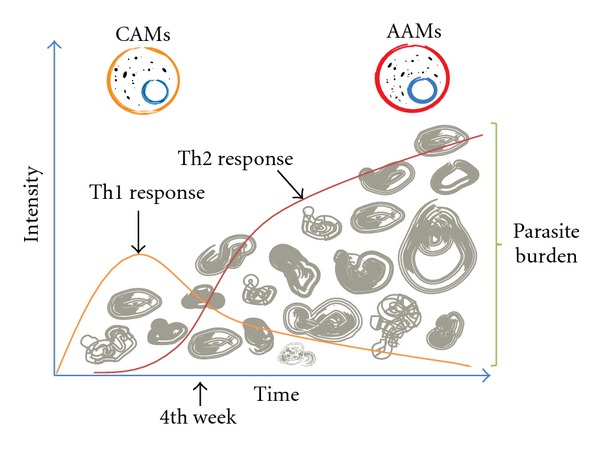
The initial Th1 response to *T. crassiceps* is rapidly replaced by a Th2 response by the third or fourth week after infection. This shift is accompanied by a change in macrophage phenotype, as the early CAM population is replaced by a dominant AAM population. Moreover, an increase in parasite burden can be observed when the AAM population and the dominant Th2 response are established.

Early studies on the immune response against this parasite were performed in the late 1970s and early 1980s by Siebert and Good [24, 25]. This work mainly focused on the humoral immune response against *T. crassiceps* and found that antibodies anti-*T. crassiceps* cannot be correlated with cytotoxic effects or tegument degradation. Later, following the definition of the dichotomous Th1 and Th2 responses, a new series of investigations were conducted by different groups. Most of these studies coincided with the general knowledge that, during the acute stage, murine infection with this parasite leads to the induction of a transient Th1 proinflammatory immune response with high serum levels of gamma interferon (IFN-*γ*), nitric oxide (NO), and IgG2a that lasts for the first 2-3 weeks and then is replaced by a dominant Th2-type response rich in IL-4/IL-13, as well as IgG1 and IgE antibodies that last for at least two months (Figure 1) [26]. Later findings demonstrated that spleen cells from *T. crassiceps*-infected mice were refractory to polyclonal stimuli such as Concanavalin A and anti-CD3 [27], indicating that infection has a clear modulatory effect on the hosts immune system. Our next studies, conducted in the late 1990s, sought to block the cytokines involved in immune regulation *in vivo* early during infection, such as IFN-*γ*, IL-4, and IL-10, or inject IFN-*γ* plus IL-2 to support our idea that a Th1 response was efficacious at eliminating the larval stage of *T. crassiceps*. Early blockade of IFN-*γ* with specific antibodies in the first week of infection greatly favored the establishment of the parasite. In contrast, the injection of recombinant murine IFN-*γ* plus IL-2 at the time point improved resistance to the infection, whereas the blockade of IL-10 or IL-4 had little effect on parasite loads [28]. 

Our proposal that a Th1 response was involved in eliminating a helminth infection was not widely accepted for several more years. Early in the 2000s, confirmatory experiments were performed with knockout mice to show that susceptibility to the larval stage of *T. crassiceps* is dependent upon signal transducer and activator of transcription-6 (STAT6) signaling, a key transcription factor involved in Th2 lymphocyte differentiation and alternative macrophage activation [29]. Conversely, resistance to this parasite was shown to be dependent on the IL-12/STAT4 signaling axis [30, 31], which is the main inducer of Th1 immunity. Thus, a Th2-type response was found to be associated with susceptibility to helminth infection, whereas a Th1-type response (dependent on STAT4) was shown to be clearly involved in protection against *T. crassiceps* (Figure 1). Moreover, when the immune responses in susceptible (BALB/c) and resistant (C57BL/6) mouse strains were compared, it was found that the Th1 immune responses mounted by C57BL/6 mice are stronger than those of susceptible mice [32]. These data together sustain the notion that susceptibility to the *T. crassiceps* metacestode is dependent upon a Th2 immune response, while resistance depends on the adequate and rapid development of a proinflammatory response.

**Figure 2:**
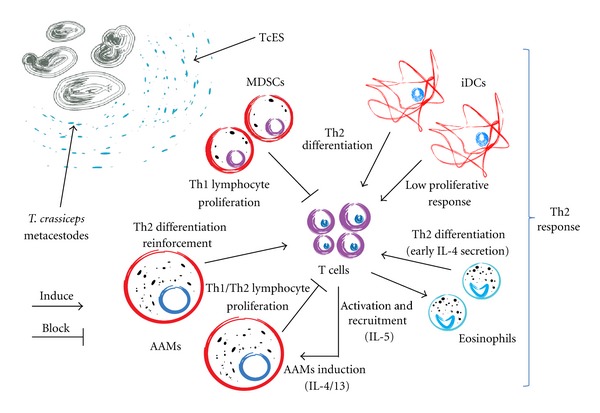
With our information to date, we propose a model of Th2 induction in which the excreted/secreted antigens of *T. crassiceps* (TcES) recruit MDSC populations to downmodulate the early Th1 response, while iDCs and eosinophils recruited by these antigens induce Th2 differentiation. AAMs reinforce Th2 lymphocyte differentiation while limiting their proliferation. Together, these changes favor a dominant Th2 response.

One of the most interesting findings from a separate series of studies was the fact that, in parallel to the shift from a Th1-type towards a Th2-type response, a distinct population of macrophages emerges; these macrophages display low IL-1*β*, IL-12, and nitric oxide (NO) secretion but express high levels of arginase-1 (Arg1), Ym1, resistin-like molecule-alpha (RELM*α*), macrophage mannose receptor (MMR), and interleukin-4 receptor alpha (IL-4R*α*). This population has a poor ability to induce antigen-specific proliferative responses in T cells and a Th2 driving ability [33] and is now recognized as AAMs (Figure 2);importantly, AAMs have been reported to be present during most helminth infections [3]. Interestingly, the immune polarization toward a Th2 profile and the establishment of an AAM population are accompanied by an increase in parasite burden during experimental murine cysticercosis caused by *T. crassiceps *[29] (Figure 1). These findings suggest that the parasite itself may be the main impetus for this tolerogenic response. 

## 3. Alternatively Activated Macrophages and Their Role in *T. crassiceps* Susceptibility and Immunoregulation 

Two main macrophage phenotypes have been described according to the inflammatory stimuli that induce their activation. Classically activated macrophages (CAMs) are activated through toll-like receptor (TLR) stimulation with bacterial-, virus- and protozoan-derived molecules such as lipopolysaccharide (LPS) and peptidoglycan as well as IFN-*γ*, tumor necrosis factor-alpha (TNF-*α*) and IL-1*β*, which are secreted during inflammatory responses. CAMs show enhanced phagocytic, microbicidal, and Th1/Th17-driving abilities and consequently have an important role in immunity to intracellular pathogens. They typically express inducible nitric oxide synthase (iNOS), which is the main enzyme involved in NO production, and they also secrete proinflammatory cytokines such as IL-1*β*, IL-12, IL-23, and TNF-*α* [38]. In contrast, AAMs are induced mainly by IL-4 and IL-13 [39] stimulation through IL-4R*α* [40], causing the activation and nuclear translocation of STAT6 [41]. Additionally, several helminth antigens have been proven to induce the alternative activation of macrophages independently of IL-4 stimuli [9–11, 42]. 

AAMs may secrete high levels of IL-10 and TGF-*β* but low levels of proinflammatory cytokines and express the enzyme Arg-1, which competes with higher affinity than iNOS for the common substrate L-arginine and produces urea, polyamines, and L-ornithine. AAMs also express YM-1, RELM*α*, programmed death-ligand 1 (PD-L1), and PD-L2 [3] and play a role in several aspects of the immune response, such as lymphocyte Th2 differentiation [33, 43], recruitment of IL-4-producing eosinophils [44], and, primarily, induction of low proliferative T cell responses [45, 46] (Figure 2). 

In recent years, it has been demonstrated that AAMs are a common cell population induced during diverse helminth infections [3, 6], in which they have been shown to display diverse roles in host survival [2] as well as resistance to this type of infection [47] or as part of the wound healing machinery [2]. However, the role for AAMs in the *T. crassiceps* cysticercosis model is quite different. 

The notion that IFN-*γ* [28] and STAT6 deficiency [29] correlate with resistance to infection led us to investigate the role of CAMs and AAMs in the immune response and susceptibility to *T. crassiceps*. Interestingly, macrophages from infected resistant mice displayed a greater ability to induce T cell proliferative responses, secreted pro-inflammatory cytokines and produced more NO, thereby displaying a classical activation phenotype, while macrophages from a susceptible mouse strain did the opposite and displayed an alternative phenotype [32]. We believe that macrophages that are recruited or polarized to become AAMs during *T. crassiceps* infection have been one of the most characterized during helminth infections. These AAMs show an increased expression or production of Arg-1, Ym-1, RELM-*α*, TREM-2, SLAM, MMR, mMGL, OX40-ligand, MHC-II, CD23, CCR5, IFN-*γ*R, IL-4R*α*, TLR4, PD-L1, PD-L2, PGE2, IL-10, and IL-6. In contrast, these AAMs have a low production or expression of iNOS, IL-12, IL-15, IL-18, IL-23, IL-1*β*, TNF-*α*, MIF, and NO [30, 32, 33, 45]. Thus, a total of 28 different molecules have been identified as altered in macrophages during experimental cysticercosis. Importantly, these molecules are intimately bound to the modulation of the immune response. Therefore, the study of such a cell population has become essential to understand helminth immunology. 

To gain insights into the role of macrophages in facilitating or clearing *T. crassiceps* infection, we developed new experimental strategies. The treatment of STAT6 KO mice, which develop CAMs and are highly resistant to infection, with an iNOS inhibitor *in vivo* rendered these mice susceptible to *T. crassiceps* infection [52]. Similarly, deficiency in TLR2, which helps to induce pro-inflammatory responses in mice that are otherwise genetically resistant, rendered them highly susceptible to helminth infection [53]. In contrast, the early depletion of AAMs with clodronate-loaded liposomes in susceptible BALB/c mice reduced parasite loads by 90% [54]. Together, these data demonstrate that AAMs, a cell population that plays a key role in immunomodulation during *T. crassiceps* infection, may also be implicated in susceptibility to this parasite, while CAMs appear to be related to resistance. 

The mechanism by which AAMs mediate susceptibility to this cestode is not currently well known, but it may be the inhibition of NO production through the expression of Arg-1 [47, 52], the release of prostaglandin E2, which also has immune-modulatory properties [55], or their suppressive capacity over lymphocyte proliferation [45]. Regardless, it is clear that the presence of CAMs is an important factor that contributes to host resistance. Several major findings support this idea, including the discovery that strains of mice that are resistant to *T. crassiceps *infection do not develop AAMs [32]; for example, C57BL/6 mice challenged with a similar number of metacestodes as BALB/c mice develop CAMs, but if STAT4-KO mice on the same resistant genetic background are similarly challenged, they have huge parasite burdens and develop AAMs [30]. In contrast, mice with a susceptible genetic background, such as BALB/c, but lacking the STAT6 gene became highly resistant to infection and do not develop AAMs. Instead, they recruit CAMs that highly produce NO, TNF-*α*, and IL-12 [29]. Moreover, as we stated above, the *in vivo *inhibition of iNOS was shown to induce susceptibility in STAT6-KO mice [52]. Thus, the activation state of macrophages plays a critical role in the outcome of helminth infection. 

Additionally, both low proliferative responses and low lymphocyte counts in tissues near the parasite may be important factors in susceptibility to this infection, as it has been shown that there are many apoptotic lymphocytes surrounding viable metacestodes in *T. solium*-infected pigs [56] and during *T. crassiceps *infection can be seen a lower lymphocyte proliferative response in susceptible mice strains than in resistant ones [27]. These findings are in line with our observations, in which we have found that AAMs induced by *T. crassiceps* can suppress the proliferative responses of naive T cells stimulated with anti-CD3/CD28 antibodies *in vitro* [45]. As we had previously detected high levels of PD-L1 and PD-L2 expression in AAMs recruited during *T. crassiceps* infection, we hypothesized that the Programmed death-1/Programmed death-Ligands (PD-1/PD-Ls) pathway may be involved in such inhibition. Thus, transwell assays and *in vitro* blockade of PD-L1 or PD-L2 were found to reverse the suppressive activity of these AAMs. Moreover, AAMs induced by *T. crassiceps* infection were also demonstrated to suppress the specific response of CD4^+^ DO11•10 cells to OVA peptide stimulation when unpolarized macrophages were used as antigen presenting cells. Again, in this assay, the blockade of the PD-1/PD-L's pathway reestablished the peptide-specific proliferative response of CD4^+^ DO11•10 cells [45]. Therefore, AAMs can participate as a third party suppressive cell. This idea was confirmed with a different set of experiments, in which we demonstrated that the presence of AAMs in a DC-mediated mixed lymphocyte reaction was sufficient to inhibit the response of CD4^+^ cells from a different genetic background. Mechanistically, AAMs recruited during chronic *T. crassiceps* infection are able to suppress immunological events mediated through distinct molecular pathways that may induce strong proinflammatory responses (Figure 2). We also demonstrated that the PD-1/PD-L pathway participates in modulating the anti-*Taenia*-specific cell proliferative response. However, whether these T cells exposed to AAMs undergo anergy and/or apoptosis and the *in vivo* significance of the PD-1/PD-L pathway in susceptibility to *T. crassiceps* are currently unknown, and further research is needed to resolve these questions. 

## 4. Immunoregulation by *T. crassiceps* Antigens

It is commonly accepted that the inhibition of proinflammatory responses and the induction of Th2 immunity during helminth infections are dependent upon the parasite's ability to excrete/secrete antigens with immunoregulatory properties that have important effects on myeloid-derived suppressor cells (MDSCs), eosinophil and basophil recruitment, DC maturation impairment, alternative macrophage activation, impaired lymphocyte proliferative responses, and, in some cases, Treg induction [8–12, 57, 58]. The first *in vivo* evidence for these conclusions is that the pharmacological treatment of helminth-infected patients can trigger pro-inflammatory responses [59] and that much experimental data have been obtained indicating that the inoculation of helminth antigens alone has the ability to induce such immunoregulatory effects, as reviewed in [12, 58]. Thus, it has been largely accepted that the *in vivo* injection of some helminth-derived antigens is able to mimic some of the immune features induced by these parasitic infections, but the mechanisms, putative receptors, and intracellular signaling pathways involved in these effects still have yet to be recognized [60]. Pioneering studies by the group led by Donald Harn at Harvard University have demonstrated that the main Th2-inducing activity of injected soluble egg antigen (SEA) from *Schistosoma mansoni* is dependent on the intact structure of carbohydrates in the antigen [61]. Thus, it was hypothesized that glycoproteins are essential for Th2 induction during schistosomiasis. This idea was rapidly adopted by several “helminth immunologists,” who corroborated many of the Harn's group findings using different sources of helminth antigens, such as *Brugia, Echinococcus, Ascaris, Caenorhabditis, Hymenolepis* [62] and, of course, *Taenia*. In this area, our team has also evaluated the effects of the *in vivo* inoculation of antigens derived from *T. crassiceps *metacestodes. The injection of a soluble extract of these larvae can rapidly (18 h post-inoculation) recruit a CD11b^+^F4/80^+^Gr1^+^ cell population, consisting of what are now called myeloid-derived suppressor cells (MDSCs), which possess a strong capacity to inhibit proliferative responses in activated lymphocytes and may have an important role in inhibiting the initial Th1 response to this parasite (Figure 2) [63]. Interestingly, when *T. crassiceps *antigens are treated with sodium metaperiodate to alter glycan structures, these antigens lose the ability to recruit MDSCs, indicating a critical role for glycoproteins in modulating the immune response to this parasite. Further research has demonstrated that glycans present in *T. crassiceps* excreted/secreted (TcES) molecules are also important in modulating DC maturation [64]. 

DC maturation involves the upregulation of several costimulatory molecules that play important roles in antigen presentation and T cell activation, such as CD40, CD80, CD86 and major histocompatibility complex II (MHCII), as well as proinflammatory cytokines such as IL-12 and IL-18. A fully mature DC is capable to induce T cell activation, proliferation, and differentiation into the Th1 phenotype, whereas an iDC drives Th2 differentiation and induces an impaired proliferative response in T cells [71]. Recently, we have found that the *in vitro* exposure of murine [64] and human [72] DCs to TcES impairs their maturation. DCs also become refractory to stimulation with different TLR ligands and thereby produce low levels of pro-inflammatory cytokines such as IL-12, IL-15, and TNF-*α*. Importantly, when these DCs exposed to TcES are used as antigen presenting cells, they are able to induce the Th2 differentiation of naïve CD4^+^ T cells (Figure 2). Moreover, all these effects of TcES are glycan-dependent [64]. Interestingly, other research groups in this field have found many similarities in the responses of monocyte-derived dendritic cells following exposure to glycoproteins derived from *Echinococcus granulosus, E. multilocularis *[73, 74], egg carbohydrate antigens from *S. mansoni *[75], or larval carbohydrate antigen from the nematode *Trichostrongylid* [76]. 

Moreover, *in vivo* assays have also demonstrated that carbohydrates in helminth-derived antigens are essential to bias Th2-type responses to bystander antigens, and thus glycoproteins from SEA and from *T. crassiceps* coinjected with the unrelated proteins human serum albumin and ovalbumin, respectively, into mice were shown to induce strong Th2 responses to these antigens. However, when glycan structures were altered, the Th2 polarization effect of the helminth antigens was eliminated [8, 61]. Thus, it is clear that host immune responses to helminth parasitic diseases or to bystander antigens are modulated by helminth-expressed glycans, and therefore most of these effects must be mediated by carbohydrate recognition receptors. It is important to keep in mind that the chemical composition of helminth antigens varies greatly among species, but the most common types are proteases, protease inhibitors, cytokine/chemokine homologs, antioxidant enzymes, lectins, and other carbohydrates [12]. Consequently, other receptors may also be involved in recognizing such diverse molecules. Likewise, it is critical to elucidate the protein, glycan, and lipid composition of helminth-derived molecules with immunomodulatory ability.

## 5. The Therapeutic Potential of *T. crassiceps*

The last two decades have witnessed a dramatic increase in the number of new cases of inflammatory diseases in developed countries, while, at the same time, the hygiene conditions in these countries have greatly improved, leading to a reduction in the prevalence of different bacterial or parasitic infections, including helminth infections [77]. Taken together, these observations led to the postulation of the hygiene hypothesis, which states that in the absence of intense infections that modulate host immunity to a Th2-type response (such as during helminth infections), the immune system then tends to present exaggerated Th1 inflammatory responses directed against microbial antigens or even autoantigens, thus leading to autoimmunity [57]. Although the contribution of genetic factors in the development of these diseases is evident, epidemiological [77–79] and experimental [80] evidence suggests that environmental factors can also be involved in the etiology of autoimmunity. 

The main experimental evidence supporting the hygiene hypothesis came from studies in which helminth-infected mice were able to successfully control type 1 diabetes (T1D) [17, 51], experimental autoimmune encephalomyelitis (EAE), an animal model of multiple sclerosis (MS) [13, 16], inflammatory bowel disease (IBD) [81], and rheumatoid arthritis (RA) [66]. More importantly, some studies conducted with parasite-derived antigens showed their ability to improve the outcomes of these diseases [14, 51, 82, 83]. Helminth therapy and its likely benefits have started to be applied in humans; the treatment of patients with the eggs of the nonhuman parasite *Trichuris suis*, which is related to the human parasite *T. trichuria*, has been shown to moderately improve the outcome of MS [84], Crohn's disease [85], and, at a lower level, ulcerative colitis [85]. Because treatment with living organisms can generate adverse or side effects [84], treatment with helminth-derived immunomodulators is a very promising alternative [58].

Based on the notion that *T. crassiceps* induces strong anti-inflammatory and long-lasting Th2 responses, characterized by high systemic levels of IL-4, IL-10, and IL-13 as well as the recruitment of different regulatory cell populations such as AAMs, MDSCs, and iDCs accompanied by low T cell proliferative responses and the induction of low NO, IL-1*β*, IL-12, IL-15, IL-18, IL-23, TNF-*α*, and IFN-*γ* levels that may block pathologic inflammation, we investigated the role of this infection in the modulation and outcome of experimental autoimmune diseases such as EAE, rheumatoid arthritis, and T1D.

Recent findings in our laboratory show that the preinfection (8 weeks) of mice with *T. crassiceps* metacestodes can reduce the incidence of EAE by 50% and reduce the severity score of the disease (1 out of 5) in sick animals. This effect was accompanied by high systemic levels of IL-4, IL-10, IgG1, and IgE and low levels of IFN-*γ*, TNF-*α*, IL-17, and IgG2a, as well as a reduced inflammatory infiltrate into the spinal cord. Importantly, we could find AAMs with strong suppressive activity over lymphocyte proliferation and a reduced number of CD3^+^ cells entering in the brain [16]. Other populations, such as CD4^+^/CD25^+^/FoxP3^+^ Tregs, have been associated with the secretion of high levels of IL-10, thereby suppressing Th1, Th2, and Th17 responses. Tregs are likely to exert this regulatory effect on autoimmune diseases, but we have not been able to find Tregs in the brain, spleen, mesenteric lymph nodes, or peritoneal cavity of *T. crassiceps*-infected mice from susceptible or resistant strains [16, 17]. Strikingly, an examination of the brains and spleens of *T. crassiceps*-infected EAE mice using flow cytometry and rtPCR failed to reveal a significant increase in CD4^+^/CD25^+^/FoxP3^+^ Treg cells [16]. 

Other research groups have shown that infection with *S. mansoni* [13], *Fasciola hepatica* [35], and *Trichinella pseudospiralis* [34] can regulate the incidence and/or severity of EAE, whereas other parasites such as *Strongyloides venezuelensis* [36] and *T. spiralis* [37, 86] did not significantly affect EAE development. Furthermore, the cytokines generally associated with the downregulation of EAE are IL-4, IL-5, and IL-10 as well as low IFN-*γ*, TNF-*α*, and IL-17, but none of these other models are associated with AAMs as possible key players in the regulation of such diseases; instead, Th2 CD4^+^ T cells are a common hallmark of EAE regulation (Table 1).

Importantly, *Hymenolepis nana, T. trichuria, Ascaris lumbricoides, Strongyloides stercolaris,* and *Enterobius vermicularis*-infected MS human patients showed a significantly lower number of disease exacerbations, brain damage, and variation in disability scores during a 4.5-year-follow up study. This protective effect was correlated with high eosinophilia, IgE titers and IL-10^+^/TGF-*β* ^+^ Treg cell induction as well as low IL-12 and IFN-*γ* secreting cells [87], partially resembling the observations in the animal models. 

Additionally, we have shown that the preinfection of mice with *T. crassiceps* can reduce the incidence of T1D induced by streptozotocin (STZ) up to 50%, mainly by lowering blood glucose levels to below 200 mg/dL, leukocyte infiltration into the pancreas and, in consequence, the degree of insulitis. Such effects last for at least 6 weeks after induction of T1D. These protective effects were associated with high systemic levels of IL-4, a reduction in TNF-*α* circulating levels, and the induction of AAM populations; however, analysis of the spleens did not show increased populations of Treg cells [16]. To our knowledge, only one work regarding STZ-induced diabetes and helminth-induced immunoregulation has been published in addition to ours; in this paper, it was shown that *S. mansoni* infection could reduce T1D incidence and pancreatic cell infiltration, but the authors did not suggest which cell populations may be involved in the modulation of this disease [48] (Table 2). 

Although other works examining the regulation of diabetes by helminth infections were primarily performed in less aggressive and slower models, such as nonobese diabetic (NOD) mice, there are several similarities and differences compared to our observations in the *T. crassiceps* model. Strikingly, it was shown that *Heligmosomoides polygyrus* infection during the early weeks of life can decrease the incidence of T1D in a mechanism dependent upon AAMs but not Tregs [15], but it has also been shown that infection with *S. mansoni* can significantly reduce the incidence of diabetes and pancreatic damage in a scenario where AAMs together with Tregs play an important role in the regulation of the disease [49]. By contrast, the infection of mice with *Litomosoides sigmodontis* promotes protection and reduced insulitis that is dependent on increased Treg populations and Th2 induction [51], while T1D incidence and blood glucose modulation in the *Trichinella spiralis* model are mainly regulated by Th2 cells [50]. Together, these studies indicate that multiple pathways are involved in the modulation of experimental T1D by helminths, but some similarities can be found regarding regulatory leucocyte populations and cytokines (Table 2).

Despite the strong regulatory activity of *T. crassiceps* in EAE and T1D, the infection with this parasite was shown to be unable to modify the outcome of experimental RA, given that 100% of infected animals developed medium clinical scores [65]. Strikingly, the pre-infection of mice with other helminths such as *Syphacia oblevata* [67] and *Hymenolepis diminuta* [70] can reduce the incidence [67] and severity [67, 70] of experimental RA. Additionally, the pre-infection of mice with *S. mansoni* [68] and *S. japonicum* [66, 69] can ameliorate RA in other models. In all of these models, the downregulation of IgG2a anti-collagen antibodies and the induction of high levels of IL-4 and IL-10 appear to be important in limiting RA progression and these effects were not achieved by *T. crassiceps* infection in this model (Table 3).

**Figure 3:**
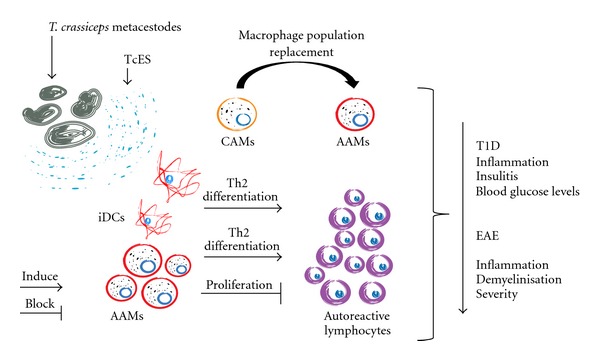
Based on our information to date, it is possible that iDCs recruited by TcES can prime Th2 differentiation, while AAMs may reinforce this activation and block pathogenic lymphocyte proliferation. Additionally, a shift from pathology-inducing CAMs to a protective AAM population can be seen, and all these changes together may protect mice from autoimmunity.

The main mechanisms involved in the abrogation of EAE and T1D with *T. crassiceps* infection may be IL-4 and IL-10 secretion as well as the induction of anergy in lymphocytes, as it has been shown that these diseases are dependent upon autoreactive lymphocyte proliferation [88, 89] and commitment to Th1 and Th17 subsets [90]. We therefore hypothesize that AAMs are the main cell population involved in tolerance induction because we have shown that they have a strong suppressive ability over lymphocyte proliferation [45] while also having the capacity to drive Th2 responses [33]. iDC populations may also be involved in this phenomenon due to their strong Th2-driving abilities, but further *in vivo* investigation is needed to confirm this hypothesis. Also, it would be important to research on the role of eosinophils [87] and B cells [91] in autoimmune disease regulation as these cells have been associated with MS regulation in humans and, at least eosinophils, are strongly and rapidly recruited by *T. crassiceps* infection [54] (Figure 3).

The absence of Treg induction during *Taenia*-induced immunomodulation of these autoimmune disease models reinforces the idea that AAMs and iDCs may play a central role in the induction of tolerance (Figure 3), but further investigation is needed to confirm the role of these cell populations in disease regulation [12, 15, 78, 79]. Additionally, we have not yet shown whether *T. crassiceps* infection can act both as a prophylactic and as a therapy option, and, more importantly, we have not yet investigated whether TcES may regulate the outcome of these diseases, which is one of the ultimate goals of our team. 

## 6. *T. crassiceps* Immunoregulation: Fibrosis and Bystander Suppression

It is commonly accepted that Th2 cytokines such as IL-4/13 and TGF-*β* induce fibrosis, which might be useful in wound healing but in other instances might be pathogenic as well [92, 93]. Moreover, AAMs have been proposed to induce fibrosis, mainly through the overexpression of Arg1, which may contribute to collagen deposition in the extracellular matrix [94, 95]. In fact, it has been shown that *T. crassiceps* infection can induce liver fibrosis in association with alternatively activated Kupffer cells and therefore exacerbate tetrachloride-induced liver damage [96]. Moreover, several epidemiological studies show that parasite-parasite coinfections are common in developing countries, with children being the most susceptible group [97–99]. Furthermore, experimental data show that helminths can modify the host immune response and alter immunity to other parasites. For example, *Litomosoides sigmodontis* infection can alter the development of a secondary infection such as *Leishmania major*, increasing susceptibility to the second parasite [100]. Similarly, it has been shown that preinfection with *T. crassiceps* modifies the immune response to *Trypanosoma cruzi* [101], *Leishmania major,* and *L. mexicana* [18], increasing susceptibility to these infections as well as tissue and organ damage resulting from the downmodulation of Th1 immunity and classical macrophage activation, which are both associated with resistance to these protozoan parasites. 

*T. crassiceps *infection can also negatively modulate the outcome of viral infections; an enhanced susceptibility to vaccinia virus via the suppression of cytotoxic T cell responses in mice infected with this helminth has been shown [102]. Moreover, the stimulation of mice with CpG, a bacteria- and virus-derived agonist of TLR9, can augment protective immunity to the cestode [103], opening the possibility for a cross regulation of susceptibility between virus and *T. crassiceps *when coinfection exists. This possibility can be extended to bacterial, protozoan, helminth, and fungal coinfections, given the discovery that TLR2 is involved in mediating resistance to this parasite [53].

## 7. Concluding Remarks

As with other helminths, infection with the cestode *T. crassiceps* induces strong and long-lasting Th2-polarized immune responses, and high systemic levels of IL-4, IL-5, IL-10, IL-13, IgG1, and IgE as well as low NO, IL-1*β*, IL-12, IL-15, IL-18, IL-23, TNF-*α*, and IFN-*γ* serum concentrations are achieved. These changes in cytokine secretion are accompanied, induced, and/or regulated by AAMs, MDSC, eosinophil and iDC populations with suppressor and Th2-driving abilities. Thus, the characteristics of the immune response to this parasite can be coopted to regulate the outcome of autoimmune diseases. In fact, we have successfully used the immune response to this parasite to regulate EAE and T1D incidence and severity. Despite these benefits, *T. crassiceps* immunoregulation has some drawbacks, such as the fact that infection with this cestode can exacerbate fibrosis and protozoa infections. Moreover, we have seen that several *T. crassiceps* antigens can mimic the effects of parasite infection, making them promising Th2 adjuvants or anti-inflammatory biocompounds that may be used in autoimmune or inflammatory disease regulation while avoiding the pathogenic side effects of infection with the live parasite. Further investigation is needed to uncover the role of TcES in the regulation or amelioration of inflammatory diseases and, in particular, the mechanisms it utilizes to modulate the immune response towards a distinct regulatory profile.

## Figures and Tables

**Table 1:** Parasite helminths involved in the regulation of EAE.

Helminth	Autoimmune disease model	Incidence	Clinical score of sick animals^¶^	Onset delay (days)	Associated leukocyte populations	Cytokines involved	Ref.
*T. crassiceps *	MOG-induced EAE	*≈*50%	0.5/5	1	AAMs, Th2 cells	High IL-4/10; low IFN*γ*, TNF*α*, IL-17	[16]
*T. pseudospiralis *	MOG-induced EAE	*≈*67%	1.5/5	11	Th2 cells	High IL-4/5/10; low IL-1*β*/6/17 IFN*γ* and TNF*α*	[34]
*S. mansoni *	MOG-induced EAE	*≈*57%	1.5/5	*≈*2	Th2 cells	High IL-4/5/10; low IL-12, IFN*γ*, TNF*α*	[13]
*F. hepatica *	MOG-induced EAE	N.S.	1/5	2	Th2 cells, Tregs, iDCs, AAMs, eosinophils and MDSCs	High TGF*β*; low IFN*γ* and IL-17	[35]
*S. venezuelensis *	MBP-induced EAE	100%	3/5	0	Non	N.S.	[36]
*T. spiralis *	SCTH-induced EAE	100%	2/4	*≈*1	Th2 cells, Tregs	High IL-4/10; low IL-17 and IFN*γ*	[37]

MOG: myelin oligodendrocyte protein; MBP: myelin basic protein; SCTH: spinal cord tissue homogenate; N.S.: not specified; ^¶^see original references, as disease severity is differentially evaluated between authors.

**Table 2:** Helminth regulation of type 1 diabetes.

Helminth	Autoimmune disease model	Incidence	Insulitis	Blood glucose level	Associated leukocyte populations	Cytokines involved	Ref.
*T. crassiceps *	T1D/MLD-STZ	*≈*50%	0%	*≈*200 mg/dL	AAMs, Th2 cells	High IL-4; low TNF-*α*	[17]
*S. mansoni *	T1D/MLD-STZ	N.S.	Relatively less infiltration and injury	*≈*200 mg/dL	Th2 cells	High IL-4/10/5; low IFN-*γ*	[48]
*S. mansoni *	T1D/NOD mice	10–30%	N.S.	<150 mg/dL	Th2 cells, eosinophils	High IL-4	[49]
*T. spiralis *	T1D/NOD mice	10%	N.S.	<200 mg/dL	Th2 cells	High IL-4	[50]
*L. sigmodontis *	T1D/NOD mice	0%^¶^	*≈*70%	≤230 mg/dL	AAMs, Th2 cells, Tregs	High IL-4/5	[51]
*H. polygyrus *	T1D/NOD mice	0%	*≈*20%	<200 mg/dL	AAMs, Th2 cells	High IL-4/13/10; low IFN-*γ*/IL-17	[15]

T1D: type 1 diabetes; MLD-STZ: multiple low doses of streptozotocin; NOD: nonobese diabetic; N.S.: not specified; ^¶^incidence defined as mice with blood glucose levels greater than 230 mg/dL, while in the other models this was defined as blood glucose levels greater than 200 mg/dL.

**Table 3:** Helminth regulation of rheumatoid arthritis.

Helminth involved	Autoimmune model	Incidence	Clinical score^¶^	Histopathology	Humoral immunity	Cytokines	Leukocytes	Ref.
*T. crassiceps *	CFA monoarthritis	100%	2/4	Similar damage in the infected group	High IgG1 and IgG2a	Low IFN-*γ* and IL-4	AAMs	[65]
*S. j* *ap* *on* *ic* um*	DBA.1 mice	60%	*≈*3/9	Ameliorated sinovial hyperplasia, mononuclear cell infiltration, and angiogenesis	Low IgG2a; high IgG1	High IL-4/10; low IL-1*β*/6 TNF-*α*, IFN-*γ*	Th2 cells, Tregs	[66]
*S. oblevata *	CFA monoarthritis	21%	1.45/4	N.S.	N.S.	N.S.	N.S.	[67]
*S. mansoni *	DBA.1 mice	0%	0/6	N.S.	Low IgG1 and IgG2a	High IL-4/10; low IFN-*γ*	Tregs	[68]
*S. j* *ap* *on* *ic* um**	DBA.1	N.S.	*≈*1/9	No synovial hyperplasia, inflammatory infiltrate, or bon/cartilage destruction	Low IgG2a	High IL-4/10 RANKL; low IL-17-*α*, IFN-*γ*, TNF-*α*	No Tregs	[69]
*H. diminuta *	CFA monoarthritis	N.S.	N.S.	Any knee swelling	N.S.	High IL-4/10; low TNF-*α*, IL-12p40	AAMs, Th2 and B2 cells, less granulocyte infiltration	[70]

CFA: complete Freund's adjuvant; *two-week preinfected mice; **seven-week preinfected mice; N.S.: not specified. ^¶^see original references, as disease severity is differentially evaluated between models.
